# Pathogenicity of reassortant H9 influenza viruses with different NA genes in mice and chickens

**DOI:** 10.1186/s13567-016-0352-y

**Published:** 2016-06-24

**Authors:** Liping Yan, Qinfang Liu, Xin Su, Qiaoyang Teng, Danqi Bao, Guangsheng Che, Hongjun Chen, Hongrui Cui, Tao Ruan, Xuesong Li, Zejun Li

**Affiliations:** Chinese Academy of Agricultural Sciences, Shanghai Veterinary Research Institute, Shanghai, China; College of Veterinary Medicine, Nanjing Agriculture University, Nanjing, China; College of Veterinary Medicine, Inner Mongolia Agricultural University, Hohhot, China

## Abstract

To better understand the influence of different NA genes on pathogenicity of H9 viruses, three reassortant H9 viruses (rH9N1, H9N2 and rH9N3) were generated and characterized. All three viruses replicated efficiently in eggs and MDCK cells, whereas the rH9N1 and rH9N3 replicated more efficiently than H9N2 in A549 cells. The rH9N3 replicated more efficiently than rH9N1 and H9N2 viruses in mice, however, rH9N3 replicated and shed less efficiently than the H9N2 virus in chickens. Further studies indicate that N3 had higher NA activity and released virus from erythrocytes faster, which may improve the adaptation of H9 influenza virus to mammals.

## Introduction, methods, and results

Influenza A viruses are enveloped viruses which contain a segmented genome of eight different negative-strand RNA molecules. Reassortment among influenza viruses may result in novel viruses that exhibit altered pathogenicity, transmissibility and even capability for interspecies transmission. There are eighteen antigenically distinct haemagglutinin (HA) and eleven neuraminidase (NA) subtypes of influenza A viruses. The H9N2 subtype is particularly noteworthy due to its widespread circulation in domestic poultry and rapid evolution by drift mutation and reassortment [1]. H9N2 subtype avian influenza viruses have been circulating in multiple avian species and sporadically infecting humans, but person-to-person transmission of H9N2 virus has been limited [2]. The H9N2 subtype was first identified in chicken farms in China in 1994 [3], since then three lineages of H9N2 viruses have circulated in poultry in Asia: G1-, Y280- and BJ-94-, represented by the prototype viruses A/quail/Hong Kong/G1/97, A/duck/Hong Kong/Y280/97, and A/chicken/Beijing/1/1994, respectively [4]. Recently, Genetic analysis of H9N2 viruses indicated that these viruses have undergone dramatic evolution. Pu et al. reported 69 different genotypes of H9N2 viruses in China from 1994 through 2013. Since 2010, one genotype, G57, has become the predominant genotype in circulation throughout China. This genotype contains genes from A/chicken/Beijing/1/1994-like, A/quail/HongKong/G1/1997-like and A/chicken/Jiangsu/1/2000 H9N2 viruses. The appearance of the G57 genotype in chickens has ended more than 10 years of co-circulation of multiple H9N2 genotypes [5]. H9N2 viruses also undergo extensive reassortment with different subtypes of avian influenza (AI) viruses including HPAI H5N1 and H7N3 viruses [6, 7]. Recently, H9N2 has contributed internal genes to H7N9 and H10N8 viruses that have caused severe human respiratory infections [8, 9].

The genes of the recent emergent zoonotic H7N9 virus originated from reassortment of H9N2, H7N3 and H7N9 subtype viruses that were linked to local live poultry markets [9]. Chickens, ducks, and environmental specimens from the markets contained heavily mixed subtypes of influenza viruses containing H5, H7, H9, N1, N2, N3, N9 [10]. The live poultry markets in China bring together many types of fowl under conditions of high density, providing an ideal environment for interspecies transmission and reassortment of avian influenza viruses (AIV). Such events may result in the generation of new strains with novel HA or NA genes. Since H9N2 virus is widespread in chickens, co-circulation with other subtype influenza viruses may result in novel H9 reassortants with different NA genes. It is well established that the HA–NA functional balance is critical for virus replication and pathogenicity [11, 12]. High genetic compatibility between H9N2 and 2009 pandemic H1N1 (pH1N1) has been reported previously [13–15]. Animal study results showed that reassortant H9N1 in the backbone of pH1N1 is more transmissible and virulent than parental H9N2 virus in pigs and ferrets, however, the reassortant H9N1 did not transmit to chickens [15, 16], suggesting that the fitness of H9 and N1/N2 affected the virus performance in different animal models. To better understand the influence of different NA genes on the pathogenicity of H9 subtype influenza viruses, we generated recombinant viruses in the backbone of H9N2 virus with different NA genes (N1, N2, N3) using reverse genetics technology. The replication and pathogenicity of these viruses in chickens and mice were then evaluated. All animal studies involved in this project were conducted in accordance with the guidelines of the Animal Care and Use Committee of Shanghai Veterinary Research Institute (Permit number: SHVRI-Po-0120).

**Figure 1:**
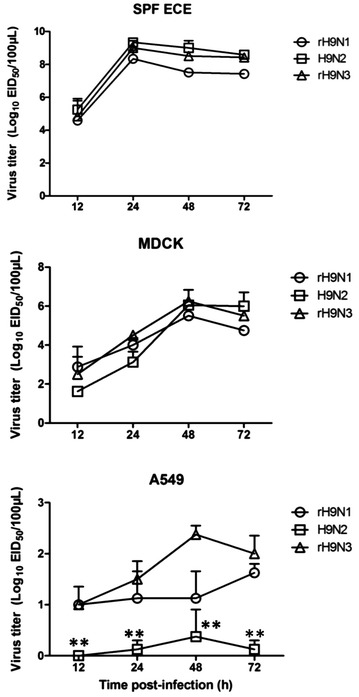
**Replication kinetics of influenza viruses in SPF ECE, MDCK and A549 cells.** Growth dynamics of recombinant viruses in SPF ECE infected at a dose of 100 EID_50_, or in MDCK and A549 cells infected at an MOI of 0.01. The virus titers are presented by log_10_ mean ± SEM EID_50_/100 mg. **p < 0.01.

A/duck/Shanghai/B2013/2012 (H9N2), isolated from a duck, was used in this study. Sequence analysis results show that this H9N2 virus belongs to the G57 genotype and contributes the internal genes to the H7N9 virus. The reverse genetics system of this H9N2 was constructed as previously described [17]. Briefly, the 8 full-length gene segments of A/duck/Shanghai/B2013/2012 (H9N2) were amplified using influenza virus universal primers as published previously [18], and cloned into the BsmBI sites of the pHW2000 vector. The constructed plasmids (pHW-H9N2-PB2, PB2, PA, H9, NP, N2, M and NS) were confirmed by sequencing. The N1 and N3 gene segments were synthetized and cloned into the BsmBI sites of the pHW2000 vector (pHW-H5N1-N1, pHW-H7N3-N3) according to the nucleotide sequence of A/duck/Eastern China/Y122/2008 (H5N1), GenBank accession No. HM370127.1; A/duck/Zhejiang/2/2011 (H7N3), GenBank accession No. JQ906581.1. Seven plasmids of H9N2 (pHW-H9N2-PB2, PB2, PA, H9, NP, M and NS) and one plasmid (pHW-H5N1-N1 or pHW-H7N3-N3) were used to generate the reassortant viruses: rH9N1 and rH9N3. All viruses were sequenced to confirm the absence of unwanted mutations and then viruses were titrated in 10-day-old specific-pathogen-free (SPF) embryonated chicken eggs (ECE) to determine the 50% embryo infectious dose titer (EID_50_) by the Reed and Muench method [19]. To check the virus replication in vitro, monolayers of MDCK cells and A549 cells were cultured in six-well plates and infected with the different viruses propagated in ECE at a multiplicity of infection (MOI) of 0.01. Twelve 10-day-old SPF ECE were inoculated with viruses at 100 EID_50_/egg. Three independent replicates were conducted for each cell line and eggs at each time point. Supernatants and allantoic fluid were collected at different time points (12, 24, 48 and 72 hpi) and titrated through inoculation of 10-day-old SPF ECE. All data were analyzed using analysis of variance (ANOVA) in GraphPad Prism version 6.0 (GraphPad software Inc, CA, USA); a p value <0.05 was considered statistically significant. The results show that wild-type H9N2 and reassortant viruses replicate efficiently in ECE and MDCK cells, and no significant difference was observed. However, in A549 cells, the reassortant rH9N3 and rH9N1 viruses grew to significantly higher titers than wild-type H9N2 (Figure 1), which suggests that the N1 and N3 genes enhance the replication of H9 viruses in human cells compared to the wild type H9N2.

To further evaluate the pathogenicity of reassortants in mammals, 50% lethal dose (LD_50_) and 50% infective dose (ID_50_) of these three viruses were tested in mice. Groups of six 5-week-old BALB/c mice were anesthetized and intranasally inoculated with serial diluted viruses (10^1^, 10^2^, 10^3^, 10^4^, 10^5^, 10^6^, and 5 × 10^6^ EID_50_) while another six native mice were inoculated with phosphate-buffered saline (PBS) (mock). To assess the ID_50_ of viruses, three of the infected mice from each group were euthanized at 4 days post inoculation (dpi) and the lung samples were collected for virus detection. To test the LD_50_ of each virus, the body weights of the remaining mice were monitored daily until 14 days after inoculation, mice that lost more than 25% of their body weight were euthanized. The results show that the LD_50_ of all three viruses were >5 × 10^6^ EID_50_, and the ID_50_ of rH9N1, H9N2 and rH9N3 were 10^4.5^, 10^5.5^ and 10^4.5^ EID_50_, respectively. This suggests that the three viruses are avirulent in mice and H9N2 is less infectious than the other two viruses.

**Figure 2:**
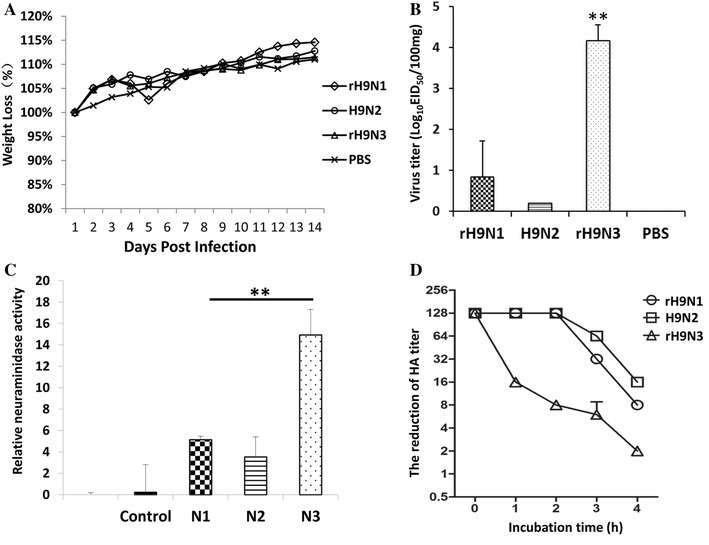
**Weight loss and replication of viruses in mice lungs (A, B); Neuraminidase activities and release efficiency of erythrocyte bound virions (C, D).** **A** Relative weight loss of mice inoculated with 10^6^ EID_50_ of virus or PBS (mock). **B** Virus titers in mice lungs. The error bars represent the standard deviations. **C** Neuraminidase activity values after normalization to renilla luciferase expression (relative activity). The activity values are displayed as mean ± SEM of three independent experiments containing three replicates. **D** Release efficiency of erythrocyte bound virions. Twofold dilutions of virus containing 1:128 HA units were incubated with equal volumes of 0.5% chicken erythrocytes at 4 °C for 1 h. The reduction in HA units was recorded for 4 h after incubation at 37 °C. **p < 0.01.

To assess virus replication of the reassortants in mammals, four groups of nine 5-week-old BALB/c mice were anesthetized and intranasally inoculated with 10^6^ EID_50_ of each virus (rH9N1, H9N2 and rH9N3) in 50 μL fresh minimum essential medium (MEM) or PBS (mock). Body weight and survival of the mice were monitored daily for 14 days after inoculation. To evaluate viral replication in the respiratory tract and other organs, three mice from each group were euthanized on 4 dpi. The lung, spleen, kidney and brain were collected for virus titration. None of the mice showed obvious clinical symptoms after infection. The mice infected with wild type and reassortant viruses showed no weight loss compared to the mock group (Figure 2A). Both rH9N3 and rH9N1 viruses replicated in the lungs of the infected mice instead of other organs (spleen, kidney and brain), while H9N2 barely replicated in the mouse lung. Notably, only rH9N3 grew to significantly higher titers in mice lungs compared to the other viruses at 4 dpi (Figure 2B). The internal genes of the H9N2, rH9N3 and rH9N1 viruses recovered from the lungs of the infected mice were sequenced and analyzed and none of the known mammalian-adaptation associated substitutions (PB2-271A, 590S, 591R, 627K, 701N etc.) were observed. All mice in the infected group seroconverted at the end of this study. Overall, the data indicate that all three viruses are able to infect mice without causing clinical signs and the N3 gene contributes to the efficient replication of rH9N3 virus in mice.

Birds are the natural host of influenza virus. In order to determine the replication and pathogenicity of wild type H9N2 and the reassortant viruses, 3-week-old SPF chickens were used in this study. A total of 36 SPF chickens were randomly allocated to four groups. Nine chickens from each group were intranasally inoculated with 10^6^ EID_50_ virus in 200 μL PBS. All birds were observed daily for clinical signs until the end of the experiment (14 days). Oropharyngeal and cloacal swabs were collected at 3, 5, and 7 dpi from the inoculated birds for the detection of virus shedding. Three chickens from each group were euthanized at 3 and 5 dpi to investigate virus replication; during necropsy, both lung and trachea were collected for virus isolation. The remaining birds were euthanized at the end of the experiment. No clinical symptoms were observed in the infected chickens during the period of the study. The wild type H9N2 and rH9N1 viruses were detected in both oropharyngeal and cloacal swabs; the H9N2 shed higher titer virus than rH9N1 (Table 1). This result indicates that the parental H9N2 and rH9N1 viruses were shed through the respiratory and intestinal tracts. However, rH9N3 was shed only through the respiratory tract. All three viruses replicated in both tracheas and lungs of the infected chickens at 3 dpi (Table 2). However, H9N2 replicated to higher titers than the other reassortants in the chicken lung while rH9N3 replicated less efficiently in both the lung and trachea (Table 2). All infected chickens showed seroconversion at the end of the experiment (Table 1).

**Table 1:** **Virus shedding and seroconversion of chickens infected with reassortant viruses on the indicated dpi**

Groups	3 dpi		5 dpi		7 dpi		Seroconversion (HI titer)
	Oropharyngeal	Cloaca	Oropharyngeal	Cloaca	Oropharyngeal	Cloaca	
rH9N1	3/3^a^ (1.58 ± 0.14^b^)	2/3^c^	2/3	3/3	1/3	0/3	3/3^d^ (245 ± 153)
H9N2	3/3 (3.33 ± 0.72)	2/3	3/3 (2.25 ± 0.66)	2/3	1/3	2/3	3/3 (512)
rH9N3	3/3 (2.25 ± 1.39)	0/3	3/3 (1.92 ± 0.52)	0/3	0/3	0/3	3/3 (1365 ± 529)

^a^The number of chickens shedding virus out of total numbers.

^b^The virus titers presented by log_10_ geometric mean ± SEM EID_50_/100 μL among positive samples.

^c^Two chickens shed virus, the titers are below the detection limit.

^d^The number of chickens that were seropositive out of total numbers on day 14 dpi, HI titers presented by geometric mean HI values ± SEM.

**Table 2:** **Virus titers in trachea or lung tissues of infected chickens**

Groups	3 dpi		5 dpi	
	Lung	Trachea	Lung	Trachea
rH9N1	1/3^a^ (4.25)	3/3 (4.5 ± 0.90^b^)	0/3	3/3 (2.5 ± 0.00)
H9N2	2/3 (3.75 ± 0.71)	3/3 (4.5 ± 0.00)	3/3 (3.58 ± 0.14)	3/3 (4.42 ± 0.14)
rH9N3	1/3 (1.5)	1/3 (3.5)	2/3 (3.25^c^)	2/3 (2.25^c^)

^a^The number of chickens positive for virus isolation out of the total number of chickens on the days indicated.

^b^The virus titers presented by log_10_ mean ± SEM EID_50_/100 mg among positive samples.

^c^Two chickens shed virus, one of them has titer below the detection limit.

All the presented data indicate that the N3 gene contributes to enhanced replication of rH9N3 in mammalian hosts rather than in chickens. Previous studies showed that NA activity plays an important role in the pathogenicity and transmissibility of influenza virus [20, 21]. To compare NA activity of different NA genes, the N1, N2 and N3 genes were cloned into *Bsm*AI and *Nhe*I sites of the pCAGGS vector. 293T cells in 6-well-plate were separately transfected with pCAGGS-N1 (1 μg), pCAGGS-N2 (1 μg) and pCAGGS-N3 (1 μg). The plasmid pCAGGS-Renilla (0.2 μg) was transfected as an internal control for the NA activity assay. The cells were incubated at 37 °C for 48 h after transfection, and then the neuraminidase activity in cell lysis was measured using the NA-XTD™ Influenza Neuraminidase Assay Kit (Applied Biosystems) according to the manufacturer’s instructions. The luminescence was measured on a SpectraMax instrument (Molecular Devices, USA). The N3 showed significantly higher NA activity than the N1 and N2. The N2 had the lowest activity compared with N1 and N3 (Figure 2C). Usually, NA activity correlates with virus release from the infected cell. To confirm the NA activity data, we compared the ability of different NA to release erythrocyte-bound virions by performing hemagglutination at 4 °C followed by incubation of the HA microtiter plates at 37 °C. The bound virions will be released due to the viral NA activity; the reduction of HA titers was recorded [22]. Briefly, fifty microliters of two-fold dilutions of the virus containing the HA titers of 1:128 was incubated with 50 µL of 0.5% chicken erythrocytes in microtiter plates at 4 °C for 1 h. The HA microtiter plates were then incubated at 37 °C for 4 h, and the reduction of HA titers was recorded every hour. The rH9N3 strain showed a 64-fold reduction in HA titer after 4 h of incubation at 37 °C, whereas the H9N2 and rH9N1 showed only 8- and 16-fold reduction (Figure 2D). Comparison of the elution properties of the reassortants revealed high correlation between NA activity and the ability of the NA to release virions from erythrocytes.

Previous studies indicated that the NA activity plays a critical role in the pathogenicity of influenza viruses; high NA activity contributed to the replication and transmissibility of 2009 pandemic H1N1 virus in ferrets [20, 23, 24]. In this study, the presented data also indicate that the rH9N3 with high NA activity replicated more efficiently in mice and in A549 cells compared with rH9N1 and H9N2, suggesting that NA activity is critical for the adaptation of AIV in mammalian hosts, which was in agreement with the previous studies [20, 23, 24]. However, in chickens, the rH9N3 replicated less efficiently than the others in both the lung and trachea, suggesting that high NA activity did not contribute to the adaptation of rH9N3 in chickens. The rH9N1 strain replicated well in the upper respiratory tract but not in the lungs, while H9N2 replicated in both upper and lower respiratory tracts. All these data suggest that the combination of H9 and N2 has better fitness in chickens. Although the rH9N1 and rH9N3 genotypes have rarely been isolated, introduction of NA genes with high NA activity may favor the H9 viruses to adapt to mammalian hosts.

## Discussion

The H9N2 used in this study belongs to the G57 genotype circulating in China. This genotype of the H9N2 virus, containing a Q226L mutation in HA, exhibits high affinity to human α-2, 6 sialic acid receptors [1]. Phylogenetic analyses showed that the G57 genotype H9N2 virus contributes internal genes to the novel H7N9 viruses, which have caused severe human respiratory infections in China [5, 25]. All data indicate that H9 subtype AIV might pose a threat to public health since both the HA and internal genes are adaptive to mammalian hosts [1]. Both rH9N1 and rH9N3 reassortant viruses had increased infectivity in mice compared with the wild type H9N2 virus, indicating that the H9 subtype AIV could gain better fitness in a mammalian host through obtainment of a novel NA gene with high NA activity. Co-circulation of H9N2 and other subtype influenza viruses increases the reassortment possibility and novel H9 reassortants with a specific NA have the potential to infect mammalian hosts. This finding justifies and warrants continuous surveillance of H9 influenza viruses, especially in areas where multiple influenza subtypes have been reported.

## Abbreviations

HA: haemagglutinin
NA: neuraminidase
LPAI: low pathogenic avian influenza
HPAI: high pathogenic avian influenza
ECE: embryonated chicken eggs
AI: avian influenza
AIV: avian influenza viruses
EID_50_: 50% embryo infectious dose titer
MOI: multiplicity of infection
SPF: specific-pathogen-free
